# Can community health worker home visiting improve care-seeking and maternal and newborn care practices in fragile states such as Afghanistan? A population-based intervention study

**DOI:** 10.1186/s12916-018-1092-9

**Published:** 2018-07-09

**Authors:** Karen M. Edmond, Khaksar Yousufi, Zelaikha Anwari, Sayed Masoud Sadat, Shah Mansoor Staniczai, Ariel Higgins-Steele, Alexandra L. Bellows, Emily R. Smith

**Affiliations:** 1 UNICEF Afghanistan, UNOCA, Jalalabad Road, Kabul, Afghanistan; 2 Ministry of Public Health, Reproductive, Maternal, Newborn, Child and Adolescent Directorate, Kabul, Afghanistan; 3 Ministry of Public Health, Community Based Health Care Department, Kabul, Afghanistan; 4 Save The Children, Kabul, Afghanistan; 5 Department of Global Health and Population, Harvard TH Chan School of Public Health, Boston, USA

**Keywords:** Community, maternal, newborn

## Abstract

**Background:**

The effects of community health worker (CHW) home visiting during the antenatal and postnatal periods in fragile- and conflicted-affected countries such as Afghanistan are not known.

**Methods:**

We conducted a non-randomised population-based intervention study from March 2015 to February 2016. Two intervention and two control districts were selected.

All female CHWs in the intervention districts were trained to provide eight home visits and behaviour change communication messages from pregnancy to 28 days postpartum. The primary outcome was the proportion of women who reported delivering in a health facility. Secondary outcomes were the proportion of women who reported attending a health facility for at least one antenatal and one postnatal visit. Outcomes were analysed at 12 months using multivariable difference-in-difference linear regression models adjusted for clustering.

**Results:**

Overall, 289 female CHWs in the intervention districts performed home visits and 1407 eligible women (less than 12 months postpartum) at baseline and 1320 endline women provided outcome data (94% response rate). Facility delivery increased in intervention villages by 8.2% and decreased in the control villages by 6.3% (adjusted mean difference (AMD) 11.0%, 95% confidence interval (CI) 4.0–18.0%, *p* = 0.002). Attendance for at least one antenatal care visit (AMD 10.5%, 95% CI 4.2–16.9%, *p* = 0.001) and postnatal care visit (AMD 7.2%, 95% CI 0.2–14.2%, *p* = 0.040) increased in the intervention compared to the control districts.

**Conclusions:**

CHW home visiting during the antenatal and postnatal periods can improve health service use in fragile- and conflict-affected countries. Commitment to scale-up from Ministries and donors is now needed.

**Trial registration:**

This trial was retrospectively registered at the Australian and New Zealand Clinical Trial Registry (ACTRN12618000609257).

## Background

There have been substantial improvements in the provision of maternal and newborn health (MCH) services in Afghanistan in recent years [1]. However, key indicators, such as receipt of any antenatal care (ANC) (59%) or postnatal care (PNC) for newborns (9%), remain low [2]. Afghanistan continues to have a fragile and challenging environment for women wishing to obtain healthcare, with maternal and newborn mortality rates remaining among the highest globally [2–4]. Reasons for this include persistent levels of violence and conflict, which limit the ability of many mothers and families to leave the home to receive health and social care [5–7]. Other important barriers are poverty, the mountainous terrain and lack of decision-making ‘power’ of women within the family [2].

Studies over the last 5 years in poor communities in south Asia and Africa have conclusively shown that community health worker (CHW) home visiting during the antenatal and postnatal periods can improve both the demand for and use of ANC, delivery and PNC services, and reduce maternal and newborn mortality by at least 15–20% [8–11]. CHWs are increasingly being ‘institutionalised’ in the workforce in south Asia and Africa through enhanced training and incentive packages [12–14].

Home visiting from female CHWs during the antenatal and postnatal periods is likely to be an important way to improve the demand for and use of MCH services in fragile and conflict-affected countries such as Afghanistan [7, 12, 15, 16]. Many CHW studies have been conducted in Africa and South Asia [8, 10]. However, to our knowledge, there have been no published studies of the effectiveness of home visiting by CHWs in conflict-affected districts or provinces.

Volunteer CHWs have been part of the health system in Afghanistan since 2001, but have not been routinely used for home visiting during the antenatal and postnatal periods [17]. In 2015, the Ministry of Public Health in Afghanistan, together with UNICEF and Save the Children, implemented a study to understand if home visiting by CHW for MCH could be effective in conflict-affected districts. The overall aim was to assess whether a CHW home visiting programme could improve care-seeking and newborn care practices in mothers living in the challenging environment of rural Afghanistan.

The primary objective was to assess the effects of the Afghanistan CHW home visiting programme on the proportion of women delivering in a health facility. Secondary outcomes were to assess the effects on the proportion of women who attended a health facility for at least one ANC and one PNC visit. In addition, we assessed effects on birth preparedness and newborn care practices, especially early initiation of exclusive breastfeeding. Effects on the knowledge of mothers about their own and their infant’s health were also assessed.

## Methods

### Study setting

This study was based in Bamyan and Kandahar provinces of Afghanistan. Bamyan is located in the central, mountainous, highland region of Afghanistan and Kandahar is in the southern, sub-desert region bordering eastern Pakistan.

The primary healthcare system includes accredited nurses and doctors who work in sub-centres, basic health centres, comprehensive health centres, and national, regional, provincial and district hospitals [17, 18]. The doctors and midwives provide all medical tests, medical examinations, and ANC, PNC and birthing services in the health facilities. The primary healthcare system also includes volunteer CHWs who work in ‘health posts’ (usually their own home in their own villages) [17]. New CHWs receive 3 months of initial training followed by annual refresher training. CHWs perform annual censuses through house-to-house visiting, which are recorded on pictorial tally sheets. Women of reproductive age are visited quarterly, new pregnancies are recorded and household listings are updated. CHWs are also trained to act as ‘civil registration vital statistics’ key informants in the villages. Village members are asked to report all pregnancies, births and deaths to the CHWs. The CHWs report the civil registration vital statistics data to their supervisors monthly. CHWs are also trained to provide family planning, maternal and child health promotion education, basic medicines (e.g. oral contraceptives, iron and folic acid, antibiotics, oral rehydration solution), and to refer mothers and children with significant illness and danger signs. CHWs are not accredited to provide medical or birthing services, these must be provided by accredited doctors and midwives. The CHWs are supervised by paid community health supervisors who provide monthly village-based supportive supervision.

### Design

This was a non-randomised, population-based intervention study conducted over a 12-month period from September 2015 to August 2016 in Bamyan and Kandahar provinces. For the evaluation, the baseline survey was conducted over a 1-month period in August 2015 and the endline survey was conducted in July 2016.

**Fig. 1:**
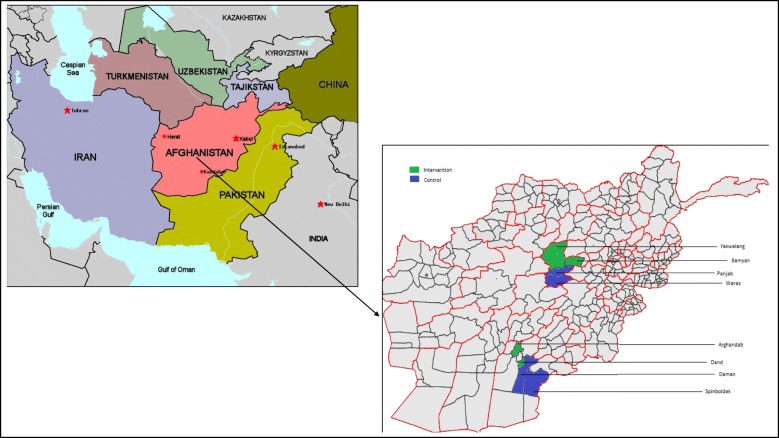
Map of Afghanistan and intervention and control districts

We used World Bank and Central Statistics Organization definitions to categorise all districts in Bamyan and Kandahar provinces into ‘conflict-affected’, ‘remote’, ‘mountainous’ and ‘poor quintile’ categories and to quantify their health services [2, 19–21]. The project team purposively selected two intervention districts per province (Arghandab, Dand, Bamyan, Yakawalang) to ensure ‘conflict-affected’ areas, ‘remote’ and ‘mountainous’ regions were represented. Two control districts per province (Speenboldak, Daman, Panjab and Waras) were then purposively chosen with similar sociodemographics, access and conflict status. Figure 1 displays the map of the study area. Tables 1 and 2 display district profile data and definitions. Intervention and control districts were similar in terms of population size, conflict risk, remoteness, mountainous terrain, wealth quintile and number of health centres. All districts had poor road infrastructure (over 50% of roads unsealed).

**Table 1:** Sociodemographic characteristics of women included in the study compared between intervention and control villages

	Number (%) of mothers in intervention villages		Number (%) of mothers in control villages	
	Baseline	Endline	Baseline	Endline
	(*n* = 709)	(*n* = 689)	(*n* = 699)	(*n* = 689)
Mother’s age (mean, SD)	27.9 (6.4)	27.8 (6.6)	28.8 (6.3)	28.4 (6.7)
Mother’s education level				
None	459 (64.7%)	460 (66.9%)	408 (58.4%)	448 (66.2%)
Primary (grade 6)	94 (13.3%)	48 (7.0%)	213 (30.5%)	53 (7.8%)
Secondary (grade 9)	27 (3.8%)	27 (3.9%)	21 (3.0%)	34 (5.0%)
High school (grade 12)	23 (3.2%)	21 (3.1%)	24 (3.4%)	28 (4.1%)
University	20 (2.8%)	10 (1.5%)	11 (1.6%)	10 (1.5%)
Madrassa	86 (12.1%)	116 (16.9%)	22 (3.2%)	92 (13.6%)
Ethnic language group				
Dari	356 (50.2%)	331 (49.8%)	353 (50.6%)	358 (52.1%)
Pashtun	353 (49.8%)	334 (50.2%)	345 (49.4%)	329 (47.9%)
Husband/partner’s education				
None	335 (47.3%)	278 (40.7%)	286 (41.1%)	360 (53.4%)
Primary (grade 6)	96 (13.5%)	80 (11.7%)	214 (30.6%)	51 (7.6%)
Secondary (grade 9)	62 (8.7%)	60 (8.8%)	57 (8.2%)	31 (4.6%)
High school (grade 12)	52 (7.3%)	56 (8.2%)	37 (5.3%)	37 (5.5%)
University	45 (6.4%)	48 (7.0%)	32 (4.6%)	37 (5.5%)
Madrassa	119 (16.8%)	165 (24.2%)	72 (10.3%)	156 (23.2%)
Husband/partner’s occupation				
Does not work	29 (4.2%)	25 (3.7%)	34 (5.0%)	19 (2.8%)
Servant/household worker	6 (0.9%)	5 (0.7%)	17 (2.5%)	10 (1.5%)
Farmer	259 (37.4%)	266 (39.2%)	229 (33.8%)	199 (20.8%)
Livestock herder	15 (2.2%)	10 (1.5%)	17 (2.5%)	18 (2.7%)
Labourer	116 (16.7%)	142 (20.9%)	144 (21.2%)	202 (30.2%)
Street seller/vendor	12 (1.7%)	22 (3.1%)	14 (2.1%)	19 (2.8%)
Shopkeeper	156 (22.5%)	103 (15.2%)	137 (20.2%)	126 (18.9%)
Businessman	18 (2.6%)	10 (1.5%)	8 (1.2%)	11 (1.7%)

**Table 2:** Sociodemographic characteristics of the intervention and control districts

Intervention or control area	Intervention	Intervention	Intervention	Intervention	Control	Control	Control	Control
Design								
Province	Bamyan	Bamyan	Kandahar	Kandahar	Bamyan	Bamyan	Kandahar	Kandahar
District	Bamyan	Yakawlang	Arghandaab	Dand	Panjab	Waras	Damaan	Speen Boldak
District population data								
Total population	55,123	57,559	69,094	83,392	53,501	84,826	15,815	88,622
Women of child-bearing age	11,198	11,870	14,356	15,678	11,456	16,743	3678	18,765
Distance in km from provincial capital	110	130	110	155	140	160	100	120
Health system characteristics								
Sub-centres	4	3	4	5	4	5	2	4
Basic health centres	3	3	3	3	3	4	2	3
Comprehensive health centres	3	2	3	4	3	4	2	3
District hospitals	1	1	1	1	1	1	1	1
Mobile health teams	1	2	0	0	2	3	0	1
Demographic characteristics								
Conflict risk^a^	Low	Medium	High	High	Low	Medium	High	High
Remote^b^	Yes	Yes	Yes	Yes	Yes	Yes	Yes	Yes
Mountainous^c^	Yes	Yes	No	No	Yes	Yes	No	No
Lowest wealth quintile^d^	81%	39%	74%	32%	65%	35%	71%	40%

^a^Conflict affected = use of armed force between warring parties in a conflict dyad, be it state based or non-state, resulting in deaths); 25 deaths or less in the previous 12 months is categorised as low intensity, 25–100 is categorised as moderate intensity and 100+ is categorised as high intensity [20, 21]

^b^Remote = District centre more than 2 h away by any form of transport from provincial capital [20, 21]

^c^Mountainous = more than 1800 km elevation at highest point of district [20, 21, 41]

^d^Lowest wealth quintile = As per the Afghanistan Living Standards Survey 2015 using Principal Components Analysis [41]

### Intervention

The intervention was a standardised training and supportive supervision package aimed at improving existing CHW capacity to provide maternal and neonatal home visits and behaviour change communication (BCC) messages from as early as possible in the antenatal period to 28 days postpartum. It was specifically targeted to female CHWs in the study area. All (100%) 289 female CHWs in the study area received the training package.

The intervention had five components. (1) Structured home visiting schedule. Each CHW was trained to provide eight home visits (four during the antenatal period and four in the postnatal period (days 1, 3, 7 and 28) and to provide health promotion and BCC messages at each visit. (2) Structured and simplified MCH BCC training ‘curriculum’. This was based on the standardised World Health Organization/UNICEF CHW training package [22]. It included key messages that the CHW should provide to mothers about birth preparedness, care-seeking for ANC, delivery care and newborn care. As most of the CHWs in Afghanistan cannot read or write, locally appropriate tools were developed and pre-tested, including pictorial counselling flip cards for birth preparedness, ANC, facility delivery, newborn greetings, PNC of mother and newborn, and maternal and newborn danger signs. Each training session lasted for 5 days and was followed by three 4-hour ‘on the job’ refresher sessions over a 12-month intervention period. (3) Structured ‘Training of Trainers’ programme. Two female government reproductive health provincial officers, one per district, were trained over a 5-day period using locally developed tools and training curriculum. The supervisors then provided training to the female CHWs as described above. (4) Supportive supervision programme. The female CHWs were visited once per month by their supervisors, who provided advice and monitoring, over a period of 12 months. These visits included both scheduled and random unscheduled visits. (5) Performance-based incentive programme. CHWs were provided with a family kit (consisting of cooking and eating utensils including pots, plates, spoons, towels and soap) (total cost approximately $21 USD per kit) for their own use at the beginning of the project if they mapped and registered pregnant women in their catchment areas. They were provided with an additional kit if the woman delivered at a health facility. Programmatic data about the number of CHWs and the intensity of their work and monitoring in the intervention districts are provided in Table 3. These data were collected by the study supervisors during their monthly supervision visits.

**Table 3:** Community health worker home visiting in intervention districts

	Bamyan	Kandahar	Total^a^
Population	112,682	152,486	265,168
Number of villages	150	131	281
Number of female CHWs	154	135	289
Population size per CHW	732	1130	918
Number of pregnant women in study area	2050	3730	5780
Number of pregnant women per CHW	13	28	20
Number (%) of women who received:			
First CHW antenatal visit	1242 (60.6%)	3436 (92.1%)	4125 (71.4%)
Second CHW antenatal visit	575 (28.0%)	393 (10.5%)	968 (16.7%)
Third CHW antenatal visit	598 (29.2%)	382 (10.2%)	980 (17.0%)
Fourth CHW antenatal visit	458 (22.3%)	272 (7.3%)	730 (12.6%)
First CHW postnatal visit day 1–2^b^	1421 (69.3%)	3029 (81.2%)	4129 (71.7%)
Second CHW postnatal visit day 3	368 (18.0%)	113 (3.0%)	481 (8.3%)
Third CHW postnatal visit day 7	330 (16.1%)	113 (3.0%)	443 (7.7%)
Fourth CHW postnatal visit day 28	198 (9.7%)	82 (2.2%)	280 (4.8%)
Total number of CHW visits in 12-month period	5180	7706	12,886
Number of visits per CHW in 12-month period	33.6	57.1	44.6
Number of visits per CHW per month	2.8	4.8	3.7
Number of visits per woman	2.5	2.1	2.2
Monitoring			
Number (%) of CHWs who received at least one supportive supervision visit	154 (100%)	135 (100%)	289 (100%)
Number (%) of CHWs who received two or more supportive supervision visits	150 (97.4%)	129 (95.6%)	279 (96.5%)

*CHW* community health worker

^a^Kandahar intervention districts: Arghandab, Dand; Bamyan intervention districts: Bamyan, Yakawalang

^b^This is a visit within the first 48 h after birth. We had exact timing data for 4393 of the 5780 mothers (76%); 966 (22%) were visited at 0–23 h and 3427 (88%) were visited at 24–47 h post birth

### Control areas

The control districts received standard care by CHWs who did not receive specific training in home visiting, MCH and BCC. All health facilities in the intervention and control areas received a health system strengthening package over the 12 months prior to the intervention delivery and throughout the intervention period. This included (1) the standard World Health Organization/UNICEF training package to midwives and neonatal nurses in essential newborn care (ENC) and Emergency Obstetric and Newborn Care and (2) training to local community health action groups on the importance of essential antenatal and newborn care.

### Data collection

Baseline and endline data were collected from women who were less than 12 months postpartum using a standardised structured survey questionnaire based on the Centers for Disease Control KPC 2000 tool [23]. Survey domains included self-reported baseline socioeconomic status, service use for ANC, delivery and PNC, birth preparedness (including seeking care for maternal postnatal complications, saving money for transportation for emergency obstetric care, pre-planning for a skilled birth attendant (SBA), arranging for a blood donor and newborn care practices (initiating breastfeeding within 1 hour of birth)), and knowledge about ANC, delivery, PNC and birth preparedness.

A separate independent team of female data collectors were trained over a 5-day period and were supervised by two independent data supervisors. The data supervisors performed spot (unscheduled) checks of approximately 10% of the surveys together with a member of the study team. An additional 10% of surveys were selected for re-survey. The data supervisors reviewed each survey form and they were not submitted for data entry until they were considered complete and without error. An additional random subsample of 10% of survey forms were also reviewed in the first month of data collection.

### Data analysis

For the outcome evaluation, we assessed mean differences in key variables from baseline (data collection immediately prior to the intervention) to endline (data collection 12 months after the intervention commenced) and compared the effects in the intervention areas compared to control areas. We randomly selected households to be visited in each district using a standard two-stage sampling method with probability of selection proportional to size (i.e. random selection of villages followed by random selection of households) [24–26]. The sampling frame was obtained from the Central Statistics Organization, which listed the names of the districts, their villages and their population size. Use of the same cooking hearth was used to define a household.

The primary outcome measure was the proportion of women who reported delivering in a health facility in the study area. Secondary outcome measures were the proportion of women who received at least one ANC visit, proportion of women who received at least one PNC visit, and the proportion of women who initiated breastfeeding within 1 h postpartum. Information on improvements in mothers’ knowledge about their own and their infant’s health was also analysed.

We calculated that we required a sample size of 1400 women to provide 80% power at a 5% significance level for the primary outcome. These calculations were based on recent data at the provincial level, which indicated that there would be a baseline level of facility delivery of 61% [2], and previous studies that indicated that there would be at least a 20% change in service use due to the intervention [8]. We also assumed that there would be one eligible woman per household. We calculated that this sample size would also provide adequate power for the secondary outcomes.

We used a multivariable ‘difference-in-differences’ approach to estimate the mean effect of the intervention on each outcome [27]. The difference-in-differences approach is based on comparing mean differences in the intervention group (before and after the intervention) to mean differences in the control group and assumes that trends in both groups are the same in the absence of the intervention [24]. Multivariable linear regression models were constructed to adjust for clustering by district and potential confounders decided a priori and to calculate adjusted mean differences (AMD), 95% confidence intervals (95% CI) and corresponding *p* values.

In the endline sample, we also assessed whether attending ANC, knowledge about birth preparedness, or sociodemographic factors were associated with facility or SBA-attended birth. Log Binomial and Poisson models with robust variances were used to estimate adjusted relative risks (adjRR), 95% CIs and corresponding *p* values [28]. SAS version 9.4 was used for all analyses.

## Results

All 289 female CHWs in the intervention districts were trained (135 in Kandahar Province (Arghandab and Dand districts) and 154 in Bamyan Province (Yakawalang and Bamyan) districts) (Table 3); 94% (2780 out of 2947) of the women in the study area agreed to participate (1408 women at baseline and 1372 women at endline). The CHWs performed 12,886 home visits to pregnant or postpartum women over the 12-month intervention period (3.7 visits per CHW per month) (Table 3). In total, 5780 pregnant or postpartum women were estimated to receive the intervention. Each CHW visited approximately 20 women and each woman received an average of 2.2 home visits throughout the antenatal and postpartum periods (mean 2.5 Bamyan, mean 2.1 Kandahar) (Table 3). Overall, 4125 (71.4%) women received a first ANC visit and 4127 (71.5%) received a visit within 48 h of birth, but only 730 (12.6%) received a fourth ANC visit and 280 (4.8%) received a fourth PNC visit on approximately day 28 postpartum.

Evaluation survey data were collected from 2780 women. Demographic characteristics were similar between intervention and control districts (Table 1). Overall, 66% of women had no education and 80% of their partners worked as farmers or labourers or were unemployed (Tables 1 and 2). Only maternal primary school education was statistically significantly different between the intervention and control groups (*p* = 0.02).

### Service use

Facility delivery increased in intervention villages by 8.2% and decreased in the control villages by 6.3% (AMD 11.0%, 95% CI 4.0–18.0%, *p* = 0.002). There were similar significant increases in the proportion of women whose delivery was attended by a SBA in the intervention areas as compared to the control areas (AMD 14.2%, 95% CI 7.3–21.2%, *p* < 0.0001) (Table 4). Attendance for at least one ANC visit improved 3.4% in intervention villages (from 71.5% to 74.9%), while ANC attendance declined in control villages (from 80.8% to 70.9%) (AMD 10.5%, 95% CI 4.2%–16.9%, *p* = 0.040). Attendance for at least one PNC visit (AMD 7.2%, 95% CI 0.2–14.2%, *p* = 0.040) increased in the intervention compared to the control districts (Table 4). The proportion of women who sought care for maternal postnatal complications increased by 14.0% in the intervention compared to the control villages (AMD 14.0%, 95% CI 4.1–23.9%, *p* = 0.006). The proportion of women who sought care for maternal antenatal complications also increased in the intervention compared to the control villages (AMD 2.8%, 95% CI –6.2 to –11.8%, *p* = 0.540), though this change was not statistically significant (Table 4).

**Table 4:** Service use and care seeking in women included in the study compared between intervention and control villages

	Number (%) of mothers in intervention villages	Number (%) of mothers in control villages	Crude mean difference (95% CI)	*p* value	Adjusted mean difference^a^ (95% CI)	*p* value
Antenatal care						
Attended at least one antenatal care visit with a skilled healthcare provider in the previous 6 months						
Baseline (*n* = 1400)	507 (71.5%)	558 (80.8%)				
Endline (*n* = 1366)	513 (74.9%)	483 (70.9%)				
Difference	3.38%	−9.82%	13.21% (6.7 to 19.7)	< 0.0001	10.54% (4.2 to 16.9)	0.001
Care seeking for antenatal or delivery complications						
Baseline (*n* = 866)	329 (75.8%)	352 (81.5%)				
Endline (*n* = 576)	229 (77.9%)	215 (76.2%)				
Difference	2.10%	−5.30%	7.33% (−1.4 to 16.0)	0.10	2.81% (−6.2 to 11.8)	0.54
Delivery care						
Facility delivery						
Baseline (*n* = 1407)	448 (63.2%)	488 (69.9%)				
Endline (*n* = 1300)	446 (71.4%)	429 (63.6%)				
Difference	8.20%	−6.30%	14.53% (7.5 to 21.6)	< 0.0001	10.97% (4.0 to 18.0)	0.002
Delivery attended by a skilled birth attendant						
Baseline (*n* = 1407)	454 (64.0%)	499 (71.5%)				
Endline (*n* = 1320)	479 (74.2%)	428 (63.5%)				
Difference	9.8%	−8.0%	18.1% (11.1 to 25.1)	< 0.0001	14.22% (7.3 to 21.2)	< 0.0001
Sought care for complications during delivery or postpartum						
Baseline (*n* = 655)	268 (82.5%)	292 (85.9%)				
Endline (*n* = 377)	170 (87.6%)	164 (89.6%)				
Difference	5.10%	3.70%	1.43% (−7.3 to 10.2)	0.75	2.36% (−6.8 to 11.5)	0.61
Postnatal care						
Attended at least one postnatal care visit with a skilled healthcare provider in the previous 6 months						
Baseline (*n* = 1392)	337 (47.9%)	369 (53.6%)				
Endline (*n* = 1309)	319 (49.6%)	300 (45.1%)				
Difference	1.7%	−8.5%	10.33% (2.8 to 17.9)	0.0072	7.20% (0.2 to 14.2)	0.04
Sought care for maternal postnatal complications						
Baseline (*n* = 739)	210 (61.4%)	302 (76.1%)				
Endline (*n* = 486)	165 (73.3%)	189 (72.4%)				
Difference	11.90%	−3.70%	15.59% (5.2 to 26.0)	0.0032	14.0% (4.05 to 23.9)	0.006

^a^Adjusted for clustering by district, maternal age, maternal education and paternal education

### Birth preparedness

The intervention appeared to have little effect on birth preparedness. The proportion of women who reported that they saved money for transportation for emergency obstetric care, pre-planned for a SBA and arranged for a blood donor was similar between intervention and control districts (Table 5).

**Table 5:** Birth preparedness and newborn care practices in women included in the study compared between intervention and control villages

		Number (%) of mothers in intervention villages	Number (%) of mothers in control villages	Crude mean difference (95% CI)	*p* value	Adjusted mean difference^a^ (95% CI)	*p* value
Birth preparedness							
Saved money for transportation for emergency obstetric care							
Baseline (*n* = 1377)		475 (67.6%)	405 (60.1%)				
Endline (*n* = 1226)		373 (60.7%)	286 (46.8%)				
Difference		−6.90%	−13.30%	6.36% (−1.1 to 13.8)	0.10	2.31% (−4.8 to 9.4)	0.53
Arranged blood donors							
Baseline (*n* = 1365)		172 (24.9%)	84 (12.4%)				
Endline (*n* = 1192)		123 (20.9%)	98 (16.3%)				
Difference		−4.00%	3.90%	−7.91% (−13.9 to –1.9)	0.0098	−9.59% (−15.4 to –3.8)	0.0013
Pre-planned for a skilled birth attendant							
Baseline (*n* = 1345)		467 (68.2%)	396 (55.9%)				
Endline (*n* = 1226)		339 (55.7%)	238 (38.6%)				
Difference		−12.50%	−17.30%	4.83% (−2.7 to 12.4)	0.21	5.32% (−2.0 to 12.7)	0.16
Breastfeeding							
Initiated breastfeeding within the first hour after birth							
Baseline (*n* = 1216)		471 (77.9%)	341 (55.8%)				
Endline (*n* = 1037)		345 (67.4%)	387 (73.7%)				
Difference		−10.50%	17.90%	−28.4% (−35.9 to –20.8)	< 0.0001	−28.22% (−35.7 to –20.8)	< 0.0001
Currently breastfeeding							
Baseline (*n* = 1385)		659 (93.2%)	640 (94.4%)				
Endline (*n* = 1366)		603 (93.8%)	631 (94.5%)				
Difference		0.6%	0.1%	0.50% (−3.1 to 4.1)	0.78	1.13% (−2.4 to 4.7)	0.39

^a^Adjusted for clustering by district, maternal age, maternal education and paternal education

### Newborn care practices

The proportion of women who initiated breastfeeding within 1 h of birth decreased in the intervention villages from 77.9% to 67.4%; however, the proportion increased in the control villages (from 55.8% to 73.7%) (AMD –28.22%, 95% CI –35.7 to −20.8%, *p* < 0.0001) (Table 5). Immediate breastfeeding rates appeared higher in home (260, 76.9%) than facility deliveries (472, 67.5%). Over 94% of women reported that they were providing breast milk to their babies in the intervention and control villages at a mean of 4 months (SD 2.5 months) postnatal in both baseline and endline samples (Table 5).

### Knowledge

In general, knowledge related to birth preparedness increased in the intervention compared to the control villages (Table 6). In particular, the proportion of women who could list at least one reason for arranging for travel around the time of birth increased by almost 9% (AMD 8.7%, 95% CI 2.9–14.0%, *p* = 0.003) (Table 6).

**Table 6:** Knowledge about birth preparedness in women included in the study compared between intervention and control villages

		Number (%) of mothers in intervention villages	Number (%) of mothers in control villages	Crude mean difference (95% CI)	*p* value	Adjusted mean difference^a^ (95% CI)	*p* value
Knowledge of importance of saving money during pregnancy							
Baseline (*n* = 1398)		605 (85.4%)	517 (74.9%)				
Endline (*n* = 1291)		509 (82.6%)	441 (65.3%)				
Difference		−2.8%	−9.6%	6.77% (0.5 to 13.0)	0.04	4.13% (−2.0 to 10.3)	0.19
Knowledge of importance of preplanning travel for birth							
Baseline (*n* = 1392)		623 (88.4%)	575 (83.7%)				
Endline (*n* = 1289)		540 (87.7%)	490 (72.8%)				
Difference		−0.7%	−10.9%	10.18% (4.6 to 15.8)	0.0004	8.46% (2.9 to 14.0)	0.003
Knowledge of importance of arranging a skilled birth attendant							
Baseline (*n* = 1390)		625 (88.7%)	572 (83.5%)				
Endline (*n* = 1285)		513 (83.7%)	502 (74.7%)				
Difference		−5.0%	−8.8%	3.84% (−1.9 to 9.5)	0.19	2.13% (−3.5 to 7.7)	0.45

^a^Adjusted for clustering by district, maternal age, maternal education and paternal education

### Predictors of service use

Maternal and paternal educational level did not appear to influence delivery at a facility or use of a SBA (Table 7). However, young mothers (< 20 years) were almost 20% more likely to give birth in a facility or with a SBA as compared to women aged 25–30 years (adjRR 1.19, 95% CI 1.05–1.34, *p* = 0.007) (Table 7). Women who attended at least one ANC visit with a skilled care provider during their pregnancy were 41% more likely to deliver at a facility or with a SBA (adjRR 1.41, 95% CI 1.27–1.58, *p* < 0.001) than women who did not (Table 7). Birth preparedness was also associated with facility or SBA-attended delivery. In particular, women who could list at least one reason why saving money was important before childbirth were 32% more likely to deliver in a facility or use a SBA (adjRR 1.32, 95% CI 1.18–1.47, *p* < 0.001) than women who could not list these reasons.

**Table 7:** Predictors of facility delivery or skilled birth attendant among women participating in the endline survey (*n* = 1376)

	Univariable model^a^		Multivariable model^a,b^	
	RR (95% CI)	*p* value	RR (95% CI)	*p* value
Maternal educational level				
No education	Reference			
Primary school	1.22 (1.09–1.36)	0.0005	1.07 (0.96–1.19)	0.21
Secondary school or higher	1.21 (1.09–1.33)	0.0003	0.99 (0.89–1.11)	0.91
Madrassa	1.10 (1.01–1.22)	0.04	1.07 (0.97–1.18)	0.20
Paternal educational level				
No education	Reference			
Primary school	1.15 (1.02–1.29)	0.02	1.08 (0.96–1.22)	0.19
Secondary school or higher	1.22 (1.12–1.33)	< 0.0001	1.07 (0.98–1.17)	0.12
Madrassa	1.01 (0.91–1.11)	0.89	0.96 (0.88–1.06)	0.46
Maternal age (years)				
15–19	1.27 (1.12–1.43)	0.0001	1.19 (1.05–1.34)	0.007
20–24	1.05 (0.96–1.16)	0.29	1.01 (0.92–1.11)	0.85
25–29	Reference			
30–34	1.09 (0.98–1.21)	0.10	1.08 (0.99–1.19)	0.10
35–40	0.98 (0.86–1.11)	0.74	1.02 (0.9–1.14)	0.80
> 40	0.97 (0.82–1.16)	0.75	1.02 (0.87–1.2)	0.76
Attended at least one antenatal care visit	1.60 (1.43–1.79)	< 0.0001	1.41 (1.27–1.58)	< 0.0001
Mothers reported having knowledge about the importance of:				
Skilled birth attendance	1.41 (1.26–1.59)	< 0.0001	1.12 (0.99–1.25)	0.06
Preplanning for travel	1.47 (1.30–1.67)	< 0.0001	1.14 (1.01–1.3)	0.04
Saving money in preparation for delivery	1.53 (1.37–1.71)	< 0.0001	1.32 (1.18–1.47)	< 0.0001

^a^Relative risk estimated by log binomial regression models

^b^Adjusted for maternal education, paternal education, maternal age, antenatal care attendance, maternal knowledge, clustering by district

## Discussion

Training of CHWs in home visiting and BCC skills during the antenatal and postnatal periods was associated with significantly improved care-seeking and service utilisation for facility delivery and ANC in the rural districts of Afghanistan included in our study. Facility delivery increased by 11%, delivery by a SBA increased by 14%, ANC attendance increased by 10% and care-seeking for antenatal and postnatal complications improved by 14% in the intervention compared to the control districts. PNC attendance was maintained in the intervention districts but declined in the control districts. Mothers’ knowledge also appeared to improve but there was little change in birth preparedness or newborn care practices.

Overall, our findings are encouraging, as the dose of our programmatic intervention was low. Each woman in our study received only two home visits from the onset of pregnancy to 28 days postpartum. Each CHW performed an average of 45 home visits over the 12-month intervention period (almost four per month); 70% of women received a first ANC visit, but only 13% of women received a fourth antenatal visit. Further, 70% of women received a visit from a CHW on the first 2 days after birth but only 8% received a PNC visit on day 7 postpartum and only 5% received a PNC on day 28 postpartum. Over the study period, the levels of conflict and insecurity in both Kandahar and Bamyan provinces increased and restricted movement of some CHWs. Some CHWs were also not allowed by other family members to visit the mothers. A particular barrier was entry to the indoor area of the home, though some CHWs were able to interact with the women in the seating area in front of their home. Competing workload priorities and other employment also restricted the time the CHWs could devote to the home visiting programme. Other challenges included the winter season limiting movement of CHWs and project staff and the dynamic nature of the conflict with new restrictions occurring on a weekly basis.

‘Dropout’ after initial engagement is a well-known part of ‘real world’ MCH service delivery [29–31]. Analyses of Demographic and Health Surveys (DHS) conducted in Pakistan, Uganda and Ethiopia describe coverage of 70–80% at the first ANC visit declining to 10–20% by the fourth visit [29, 32, 33]. Coverage of CHW home visits (50–70%) in ‘real world’ programmatic settings is also substantially lower than experimental research sites (95–100%) [8, 34, 35].

Encouragingly, our study shows that a dose as low as two home visits per pregnant woman can have an important effect on care seeking for service delivery. Increasing the dose of home visits per woman is likely to result in an even greater effect on service use and neonatal morbidity and mortality in our setting in rural Afghanistan. A clear dose response of increasing ANC and PNC service use with increasing intensity of CHW home visiting has been shown in analyses of observational data from Asia and Africa [8, 36, 37]. Clinical trial data also indicate that neonatal mortality can be reduced by up to 45% if a coverage of 90–100% of CHW home visits can be achieved [8, 35–38]. Of note, the Ministry is considering implementation of a ‘focused’ package of home visits limited to one ANC and one PNC visit, which may be more feasible to implement in our study area.

Mechanisms to increase MCH coverage must include demand generation. A recent Cochrane systematic review reported that CHW packages that included community mobilisation strategies had a 20% greater impact on coverage and health outcomes compared to CHW home visits alone [8, 10]. In our study, the Ministry of Public Health village level community health action groups were trained in intervention and control districts as part of the health system strengthening strategy. However, their activities were limited throughout the study period due to difficulties in implementing supportive supervision for them.

The intervention was made up of five components (home visits, training curriculum, training programme, supervision, incentives). Supportive supervision and in-kind payments are important for the operationalisation of home visits by volunteer CHWs [8–10, 39, 40]. Many studies have shown that cash and in-kind incentives are highly valued by a volunteer CHW workforce [8–10], yet the training stipend is also an important incentive and must be considered part of the essential intervention package [17]. Support to the CHWs through careful monthly supervision was also essential in this fragile context. However, the difficulties with monthly supervision should not be underestimated and are well described in other studies (e.g. ensuring that the most remote area CHWs are supervised, maintaining transport links and ensuring that the correct travel and accommodation per diem is paid) [8–10]. In Afghanistan, community health supervisors are paid staff members and have been embedded within the health system for the last 10 years [17, 22]. Their role is much needed and we are working with the Ministry to ensure sustainability of community health supervisor funding, training and supervision.

Overall, our baseline ANC, facility delivery, PNC and other service utilisation rates were similar to Afghanistan DHS 2015 provincial data [2], indicating that our data collection procedures worked well. The 2015 Afghanistan DHS reported slightly lower national level rates than those provided herein (national level ANC (59%), facility delivery (48%), PNC to mother (40%), PNC to infant (9%)) [2]. However, the Afghanistan DHS also reported a wide variation in coverage including rates as low as 10–20% in very poor provinces such as Nuristan and Urzugan, where attendance at community health facilities requires a longer than 4-hour walk and where the ability to leave the home is markedly restricted [2]. The modest increases reported in our study will mean much for the mothers and infants who live in similar poor and deprived districts across Afghanistan.

Mothers’ knowledge about birth preparedness (saving money for transportation, preplanning travel for facility delivery, preplanning for a SBA) appeared to be maintained in the intervention villages compared to the control villages, and also appeared to be significantly predictive of later service use. However, there appeared to be little difference between the intervention and control groups in mothers’ actual actions in saving money for transportation, preplanning travel for facility delivery or preplanning for a SBA. These findings may reflect the limited resources and financial decision-making power of the women in the deprived villages that participated in our study. Increasing the intensity and quality of CHW home visiting is needed to change the complex behaviours required for birth preparedness. This includes CHW interaction with other family members, such as fathers and grandfathers, who are the traditional decision-makers in the family setting. Other initiatives, such as cash transfers, demand side financing and provision of local transport options, are also needed [10, 39, 40].

Little improvement in neonatal care practices was observed. In particular, the proportion of women who initiated breastfeeding within 1 hour of birth declined in the intervention villages by 10%. Other studies have indicated difficulties in changing breastfeeding behaviour at both community and facility level [37, 40]. Our health system strengthening included facility-based ENC training and the importance of early initiation of breastfeeding. However, we had few resources available for ongoing supportive supervision and we were aware that quality of ENC may have been poor in both the intervention and control facilities. We also reported that breastfeeding rates appeared higher after home (260, 76.9%) than facility deliveries; thus, we feel that more facility-based work is needed in our study area. Increasing rates of referral and care seeking will not be effective in improving morbidity and mortality if health facilities cannot deal with the work load or have poor quality of care [31–33]. Interestingly, our levels of breastfeeding (94%) were maintained at high levels postpartum in both intervention and control villages at baseline and endline. These data require more investigation as to their accuracy.

Our study had some limitations. It was non-randomised and we purposively chose the intervention and control districts to ensure that both fragile and conflict-affected areas were included. However, we used adjusted difference-in-difference analyses to account for baseline differences and any potentially differing trends in the intervention and control areas [27]. Our intervention dose was low, as described above, due to implementation in difficult conflict-affected areas but we were still able to show an important difference on key outcomes. Our data collectors were allowed to spend only a short period of time in the locations to collect data due to concerns about security. Thus, our evaluation questionnaire, especially the newborn and knowledge sections, was more restricted than previously planned. Our outcome data were also all self-reported and we were not able to verify even a subsample due to project restrictions. Additionally, there was some deterioration in facility delivery, SBA and ANC coverage in the study area, likely due to increasing levels of conflict restricting access; however, longer term surveillance is needed to understand the ongoing trends.

Strengths of our study included our ability to adhere to our data quality and supportive supervision plan, which included random re-visits and verification. Other strengths included our population- and community-based implementation and evaluation. We also had a large sample size and a high response rate. Finally, definitions of ‘fragile’ and ‘conflict affected’ also vary; herein, we used clear World Bank and Organisation for Economic Co-operation and Development (OEDC) categories (‘fragile’ districts requiring external assistance for peacekeeping, governance and financial assistance, and ‘conflict affected’ districts requiring armed force resulting in battle-related deaths) [19–21], which do not include ‘natural disasters’ such as earthquakes, floods or fire.

## Conclusion

Overall, our CHW home visiting intervention appeared to improve care seeking and knowledge but not birth preparedness and newborn care practices. Our results indicate that home visiting from CHWs during the antenatal and postnatal periods is likely to be a valuable part of the health system in rural Afghanistan, but that improved training packages are also needed for more complex behaviours such as birth preparedness and newborn care. More work is also needed in strengthening of knowledge and quality of care, especially in specific essential care components including the promotion of early initiation of breastfeeding and other newborn care practices. Further system changes are also required to ensure the sustainability of the CHW programme in Afghanistan, including performance-based payments for completion of home visits and outcomes such as facility delivery. Increasing the intensity and engagement of CHW with families of pregnant and postpartum women and increasing demand generation is also important. Encouraging new findings have arisen from studies focused on cash transfers to CHWs and mothers for facility delivery and ‘mini motorcycle ambulances’ for transport of unwell pregnant mothers and their young children in Afghanistan, which will be presented in subsequent papers. Our results also indicate that CHW home visiting will be highly effective in other conflict-affected countries where women have little power to seek care for themselves or their young children. However, more studies are urgently needed especially from African states and other cultural contexts.

## Appendix

**Table 8:** Sociodemographic characteristics of women included in the study in Kandahar and Bamyan villages at baseline

	Number (%) of mothers in Bamyan intervention and control villages at baseline (*n* = 709)	Number (%) of mothers in Kandahar intervention and control villages at baseline (*n* = 699)
Mother’s age (mean, SD) (*n* = 1397)	27.8 (5.9)	28.8 (6.7)
Mother’s education level (*n* = 1408)		
None	502 (70.8%)	365 (52.2%)
Primary (grade 6)	70 (9.9%)	237 (33.9%)
Secondary (grade 9)	24 (3.4%)	24 (3.4%)
High School (grade 12)	41 (5.8%)	6 (0.9%)
University	26 (3.7%)	5 (0.7%)
Madrassa	46 (6.5%)	62 (8.9%)
Ethnic language group (*n* = 1407)		
Dari	709 (100%)	0 (0%)
Pashto	0 (0%)	699 (100%)
Husband/Partner’s education (*n* = 1408)		
None	272 (38.4%)	350 (50.1%)
Primary (grade 6)	98 (13.8%)	212 (30.3%)
Secondary (grade 9)	68 (9.6%)	51 (7.3%)
High school (grade 12)	64 (9.0%)	25 (3.6%)
University	70 (9.9%)	7 (1.0%)
Madrassa	137 (19.3%)	54 (7.7%)
Husband/Partner’s occupation (*n* = 1370)		
Does not work	30 (4.4%)	33 (4.9%)
Servant/household worker	4 (0.6%)	19 (2.8%)
Farmer	345 (50.1%)	143 (21.0%)
Livestock herder	4 (0.6%)	28 (4.1%)
Labourer	118 (17.1%)	142 (20.9%)
Street seller/vendor	2 (0.3%)	24 (3.5%)
Shopkeeper	55 (8.0%)	238 (35.0%)
Businessman	11 (1.6%)	15 (2.2%)
